# Free‐Breathing Magnetization Transfer Imaging of the Lung at 0.55 T Using bSTAR

**DOI:** 10.1002/mrm.70341

**Published:** 2026-03-12

**Authors:** Alexandra Braun, Grzegorz Bauman, Maurice Pradella, Jonathan Röcken, Katrin E. Hostettler, Oliver Bieri

**Affiliations:** ^1^ Department of Biomedical Engineering University of Basel Allschwil Switzerland; ^2^ Department of Radiology, Division of Radiological Physics University Hospital Basel Basel Switzerland; ^3^ Department of Radiology University Hospital Basel, University of Basel Basel Switzerland; ^4^ Clinics of Respiratory Medicine University Hospital Basel Basel Switzerland

**Keywords:** balanced steady‐state free precession, bSTAR, low field, lung, magnetization transfer

## Abstract

**Purpose:**

To develop a clinically applicable method for magnetization transfer (MT) imaging of the lung at 0.55 T.

**Methods:**

MT imaging of the chest was explored in healthy volunteers at 0.55 T using a self‐gated 3D half‐radial dual‐echo balanced steady‐state free precession sequence (bSTAR), a 2D multi‐slice gradient echo method (GRE) acquired in end‐expiratory breath‐hold, and a self‐gated 3D half‐radial single‐echo ultra‐short TE (UTE) approach. MT contrast relied on RF pulse prolongation for bSTAR and on pulsed off‐resonance irradiation for UTE and GRE. Data reconstruction from the self‐gated scans was performed offline and for the end‐expiratory tidal phase using a compressed sensing algorithm. MT ratio (MTR) maps were derived from an MT‐weighted and a non‐MT‐weighted scan and took 8:32 min for bSTAR, 17:16 min for UTE, and 0:48 min for GRE. In addition, patients with pulmonary diseases underwent bSTAR MTR imaging.

**Results:**

MT imaging was successfully performed in all volunteers with highly similar average MTR values for the lung of 28.8 pu (UTE), 29.4 pu (GRE), and 30.7 pu (bSTAR). In terms of resolution, however, bSTAR clearly outperformed both UTE and GRE variants. Finally, MTR imaging with bSTAR demonstrated high reproducibility in volunteers and showed substantially different MTR values for patients with various pulmonary diseases.

**Conclusion:**

At 0.55 T, MT‐sensitized bSTAR offers in vivo high‐resolution free‐breathing MTR imaging of the entire lung in clinically acceptable scan times and shows high reproducibility. Initial results suggest that MTR imaging at 0.55 T may potentially serve as a noninvasive biomarker for investigating and differentiating pulmonary diseases.

## 1 Introduction

Magnetization transfer (MT) refers to the exchange of magnetization between mobile and bound protons. The concept was initially proposed by Wolff and Balaban in the late 1980s for generating MT contrast images through saturation transfer [1]. While most tissues exhibit some degree of MT effects [2], the behavior of MT in lung tissue, a unique anatomical structure with a high air content and rapid T2*‐related signal decay, is not well understood.

To the best of our knowledge, the only demonstration of in vivo MT MRI of the human lung was by Kuzo et al. in 1995 on a 0.1 T MR‐scanner [3]. They used an interleaved non‐MT‐weighted and MT‐weighted single‐slice gradient‐echo acquisition with a total scan time of approximately 13 min. MT‐weighting was achieved using pulsed off‐resonance irradiation. Kuzo et al. investigated magnetization transfer ratio (MTR) contrast in normal lung tissue and for various parenchymal diseases. They reported no significant MT effects in normal and atelectatic lungs, for flowing blood, or acute pulmonary edema, but observed strong MT contrast in chronic parenchymal diseases. It is mentioned that MT effects appear to be proportional to the extent of interstitial fibrosis and may be useful for differentiating parenchymal abnormalities.

More recently, the feasibility of in vivo MT MRI of the lung was re‐investigated in mice on a 4.7 T scanner using a zero‐echo time approach [4]. The results of this study indicate that normal lung tissue exhibits MT effects between those of fatty tissue and liver tissue. The authors suggested a potential application of MT contrast for diffuse fibrotic lung diseases but also stated that MT MRI of the lung is unfeasible at standard clinical field strengths using conventional MRI sequences.

In summary, there is very little known about in vivo MT effects in the human lung and any technique development is challenged not only by the lung's physiological motion but also by its intrinsic low signal that accentuates further at high fields for gradient‐echo methods due to a very rapid T2*‐related signal decay [5, 6]. Only recently, however, a new generation of commercial whole‐body 0.55 T MRI systems has been introduced to the market. These clinical systems represent a modern alternative to the traditional high‐field MRI systems and have triggered a renewed widespread interest in low‐field MRI research and methods development.

Generally, with decreasing main magnetic field strength, mesoscopic susceptibility effects arising from the large alveolar air‐tissue interface and leading to the pronounced T2*‐related signal decay of parenchymal tissue at high fields can be successfully mitigated. Likewise, macroscopic field inhomogeneities are markedly reduced, which appears especially appealing for balanced steady‐state free‐precession (bSSFP); a sequence that is known to offer the highest signal‐to‐noise ratio (SNR) per unit time [7] and thus counteracts the loss in SNR with decreasing fields. In combination with a substantially shortened TR, a half‐radial dual‐echo bSSFP sequence, termed bSTAR [8, 9], has recently demonstrated excellent prospects for high‐resolution structural imaging of the lung at 0.55 T [10].

Of particular note in this context is the fact that bSSFP is very sensitive to MT [11]. In contrast to standard pulsed MT preparations with RF spoiled gradient echo (GRE) using off‐resonance irradiation, MT effects with bSSFP can be adjusted using a simple modulation of the duration of the RF pulses used for excitation [12]. Since its introduction, MT‐sensitized bSSFP has been explored for almost two decades for MTR mapping, for example, of the brain [13, 14, 15] or the heart [16], as well as for quantitative MT imaging [17, 18, 19]. Moreover, only recently, the prospects of rapid, high‐resolution, full brain MTR imaging with bSSFP have been demonstrated at 0.55 T [20].

Therefore, in this work, we propose to fuse the concepts of MT‐sensitized bSSFP with self‐navigated bSTAR for high‐resolution in vivo MTR imaging of the human lung at 0.55 T. First, the feasibility is evaluated in volunteers and the achievable MT contrast is compared to standard MT‐prepared GRE methods that are based on pulsed off‐resonance irradiation. This aims to demonstrate that MT‐sensitized bSTAR offers highly similar MTR values for the lung as in standard MT‐prepared GRE sequences but offers artifact‐free MTR images with higher spatial resolution and within clinically relevant scan times. Then, the overall reproducibility of MTR mapping of the lung with MT‐sensitized bSTAR is evaluated in a scan‐rescan experiment. Finally, its applicability and transferability to the clinical setting is demonstrated in representative patients for a range of lung diseases.

## 2 Methods

In vivo chest MRI was performed on a commercial whole‐body 0.55 T system (MAGNETOM Free.Max, Siemens Healthineers, Erlangen, Germany) equipped with a six‐channel chest array coil and a six‐channel spine coil. Subjects were positioned supine in the scanner isocenter.

### 2.1 MT Sequences

MT imaging of the lung was explored using a custom MT‐sensitized bSTAR sequence, a product MT‐prepared 2D GRE sequence, and a custom MT‐prepared ultra‐short TE (UTE) sequence, as described in detail below.

Self‐gated MT‐sensitized bSTAR [8] imaging was performed using an interleaved wobbling Archimedean spiral pole (WASP) trajectory [9]. As with bSSFP, a simple modulation of the duration of the RF excitation pulse was used to modulate MT‐weighting with bSTAR [12]: short RF pulses yield strong MT‐weighting, whereas MT‐saturation effects can be mitigated by increasing the RF pulse duration (i.e., by decreasing the RF pulse power). Generally, this will affect the TE and TR, but all other sequence parameters are kept constant for MTR imaging. In summary, the MT‐weighted bSTAR scan has a short TR (1.86 ms) and thus short acquisition time (3:06 min), whereas the non‐MT‐weighted scan has a rather long TR (3.26 ms) and prolonged scan time (5:26 min). MT‐sensitized 3D bSTAR imaging was performed with a nominal resolution of 1.9 mm isotropic using an undersampling factor of 2.3.

MT‐prepared, Cartesian, GRE imaging was performed in end‐expiratory breath‐holding due to the missing self‐ and prospective‐navigation capabilities. Thus, an ungated but interleaved 2D multi‐slice acquisition was used with a short effective TR (17.5 ms) comprising an optional module for the pulsed MT preparation (comprising a nonselective Gaussian off‐resonance irradiation pulse in combination with large spoiler gradients) followed by a module for the Cartesian GRE kernel readout (comprising a slice‐selective, Sinc‐shaped RF excitation pulse with quadratic phase increment, single echo readout, and constant crusher gradients). Both MT‐prepared GRE scans, that is, with and without pulsed off‐resonance irradiation module, had essentially the same TR and thus acquisition time (0:24 min). Imaging was performed with an in‐plane resolution of 3.5 mm and a slice thickness of 10 mm using parallel imaging (GRAPPA [21]) with an acceleration factor 2 and 24 autocalibration lines. Theoretically, only two breath‐holds are required for MTR mapping. In practice, however, consecutive breath‐holding is likely performed at slightly different breathing positions. This will lead to variations in the lung volume which in turn affects the lung density and thus the lung signal intensity; unless breath‐holding is performed incidentally at close to identical breathing positions. To this end, three MT‐weighted and three non‐MT‐weighted GRE scans were acquired to allow retrospective matching. Moreover, intermittent short breaks of about 30 s in‐between breath‐holding maneuvers were used to allow for recovery. This resulted in a substantially prolonged scan time (about 5 min) for MTR imaging with 2D MT‐prepared GRE.

Self‐gated MT‐prepared, 3D UTE [22] imaging was performed using WASP. Each UTE kernel (comprising a nonselective, low‐flip angle, RF excitation pulse with quadratic phase increment, a half‐radial centered‐out single‐echo readout with ramp sampling, subsequent readout rewinding gradients, followed by constant crusher gradients) preceded the same optional pulsed MT preparation module as used in the Cartesian 2D GRE scan. Both MT‐prepared UTE scans, that is, with and without pulsed off‐resonance irradiation module, had the same TR (18.5 ms) and thus acquisition time (8:38 min). MT‐prepared 3D UTE imaging was performed with a nominal resolution of 3.0 mm isotropic using an undersampling factor of 3.67.

For all further sequence details, see Table S1.

### 2.2 Volunteer Scans

This study included four healthy volunteers (25 years, female; 26 years, female; 30 years, female; 24 years, male). Each volunteer was scanned once for a comparison of all three proposed MT sequences. In addition, for the assessment of the intra‐subject long‐term repeatability of MTR imaging with MT‐sensitized bSTAR, each volunteer was scanned five times over a period of 9 months. The local Ethics Committee approved the study and written informed consent was obtained from all volunteers.

### 2.3 Patient Scans

To demonstrate its feasibility in a patient setting, MT‐sensitized bSTAR was part of an exploratory clinical lung MRI protocol in seven patients with known pulmonary diseases (pulmonary fibrosis [60 years, female; 67 years, female; 75 years, female; 76 years, male]; cystic fibrosis [22 years, female]; T2‐low asthma [52 years, female]; CTEPH [40 years, male]). These scans were approved by the local Ethics Committee and written informed consent was received from all patients. One patient was scanned twice, 65 days apart.

As only little is known about MT contrast in the lung, native ^1^H ventilation and perfusion MRI was also performed using a matrix pencil (MP) analysis of time‐resolved acquisitions of 2D snapshot‐like bSSFP images, as described in detail elsewhere [23]. This allowed a qualitative comparison of functional lung defects with the proposed MTR contrast images.

The patients underwent conventional lung function tests and, if possible, also received a CT scan for further analysis and comparison of the MT MRI data.

### 2.4 Image Reconstruction

Image reconstruction and post‐processing were conducted off‐line. Prior to the image reconstruction, a k‐space trajectory correction based on gradient impulse response function measurements was performed [24]. Subsequently, 3D MT‐sensitized bSTAR and 3D MT‐prepared UTE raw datasets were reconstructed using compressed sensing with a fast iterative shrinkage‐thresholding algorithm (FISTA) [25]. The L1 norm minimization was performed through adaptive data‐driven Bayesian shrinkage by means of wavelet soft‐thresholding [26]. The coil sensitivity profiles were estimated from a low‐resolution gridding‐based image reconstruction of each acquisition.

For 3D MT‐sensitized bSTAR, the data from the center‐out and center‐in readouts were reconstructed separately, and their magnitude images were subsequently summed up. To prevent signal intensity bias between the non‐MT and MT‐weighted acquisition, the coil sensitivity profile from the reconstruction of the MT‐weighted data was also used for the non‐MT‐weighted data reconstruction [20]. Further details on the reconstruction process can be found elsewhere [9].

Respiratory gating with MT‐sensitized bSTAR and MT‐prepared UTE was performed between the 0.45 and 0.95 quantiles of the full breathing cycle, corresponding to the end‐expiratory tidal phase.

Changes in the tidal breathing depth, however, may lead to small variations of the reconstructed lung volume in the end‐expiratory tidal phase which might affect lung MTR measures. To this end, for bSTAR, respiratory gating was also performed in one volunteer between the 0.05 and 0.45 quantile of the full breathing cycle, corresponding to the inspiratory tidal phase and between the 0.55 and 0.95 quantile of the full breathing cycle, corresponding to the expiratory tidal phase. In this case, the spatial isotropic resolution was reduced to 2.5 mm.

The GPU‐accelerated reconstruction pipeline was written in C++ as standalone software (GNU Compiler Collection 13.2 64‐bit on Linux operating system), CUDA Toolkit 12.8 (NVIDIA Corp., Santa Clara, CA, USA), and Insight Toolkit library 5.3 [27]. The workstation was equipped with two Epyc 7502 CPUs (AMD Inc., Santa Clara, CA, USA) and a Quadro RTX 8000 GPU (NVIDIA Corp.).

Postprocessing of the time resolved series of bSSFP images for ventilation and perfusion imaging in patients was performed offline and separately for each slice using TrueLung [28].

### 2.5 Data Analysis

All numerical analyses in this study were performed in Python 3.13.2.

Prior to MTR calculation, the reconstructed volumes in the end‐expiratory tidal phase of the self‐gated MT‐weighted and self‐gated non‐MT‐weighted scans were co‐registered to account for possible bulk motion using an adaptive graph diffusion regularization [29]. For MT‐prepared GRE, from the series of non‐MT‐weighted scans, two quasi‐co‐registered scans were chosen for MTR calculation. MTR images were expressed in percent units (pu) and calculated pixelwise using MTR = (*S*_non‐MT_ − *S*_MT_)/*S*_non‐MT_, where *S*_non‐MT_ and *S*_MT_ refer to the signal of the non‐MT‐weighted and the MT‐weighted bSTAR scan, respectively.

Masks of the lungs were created for MT‐sensitized bSTAR images using simple thresholding followed by manual refinement. From these masks, histograms of MTR values in the lung were computed for volunteers and patients. For one volunteer, an additional mask for the largest vessels was created to analyze the vascular contribution to the whole lung MTR.

## 3 Results

**FIGURE 1:**
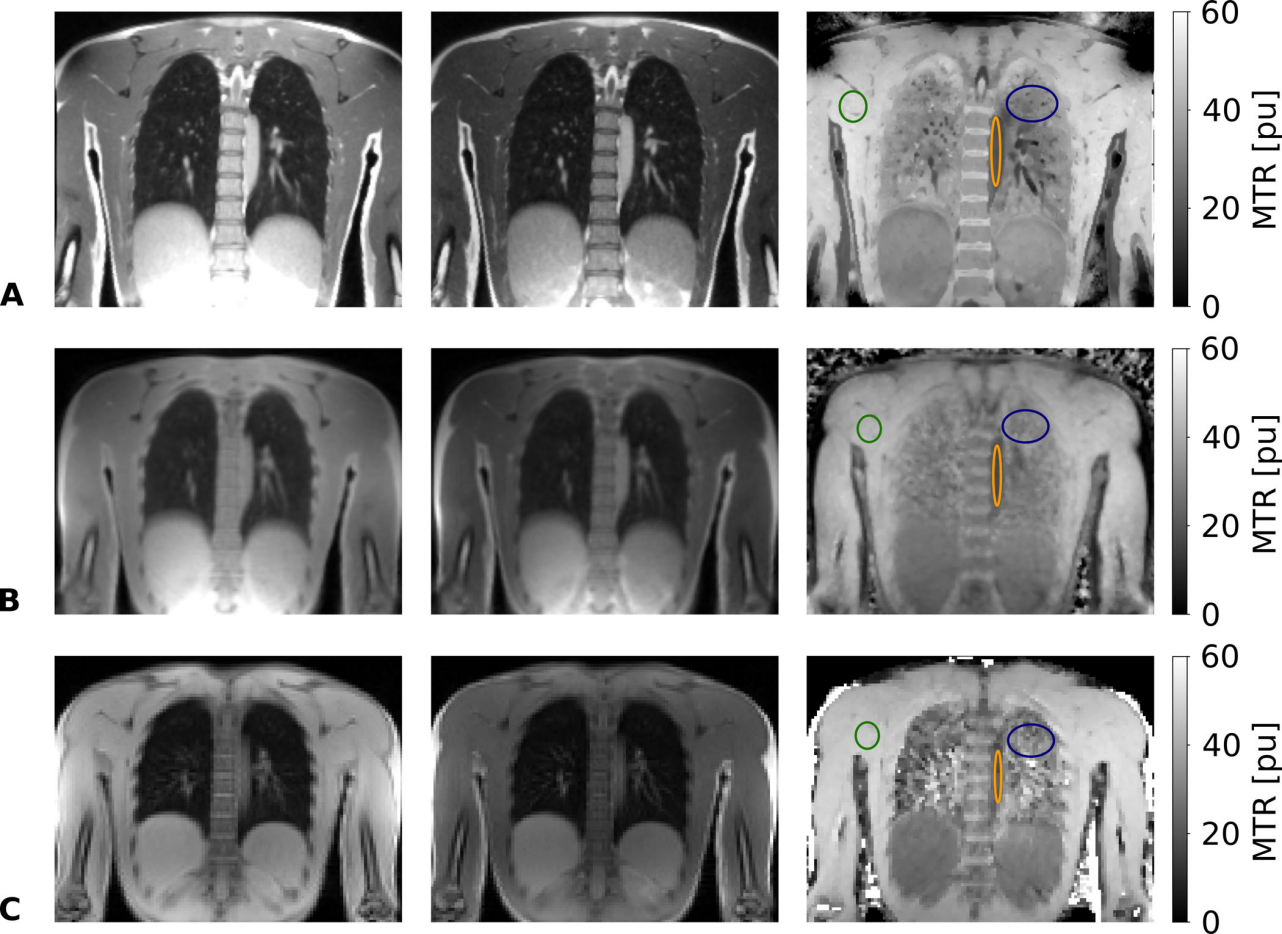
Comparison of non‐MT‐weighted (left column), MT‐weighted (middle column) and corresponding MTR image (right column) for a representative coronal slice of a healthy volunteer acquired at 0.55 T with different MT sequences. (A) 3D MT‐sensitized bSTAR (nominal resolution: 1.9 × 1.9 × 1.9 mm^3^), (B) 3D MT‐prepared UTE (nominal resolution: 3.0 × 3.0 × 3.0 mm^3^), and (C) 2D MT‐prepared GRE (in plane resolution: 3.5 × 3.5 mm^2^, 10 mm slice thickness). 3D MT‐sensitized bSTAR and 3D MT‐prepared UTE scanning was performed in free‐breathing, whereas 2D MT‐prepared GRE imaging was performed in breath‐holding. Selected regions of interest for MTR value assessment in the muscle tissue, the lung, and the blood, are indicated by the green, blue, and orange ellipses, respectively.

A representative coronal non‐MT‐weighted and MT‐weighted image of the chest together with the resulting MTR image is shown in Figure 1 for a healthy volunteer across all investigated sequence variants. Generally, MT‐sensitized bSTAR of the lung (Figure 1A) offers much better anatomical detail as compared to MT‐prepared UTE (Figure 1B) or GRE (Figure 1C). Moreover, MT‐prepared UTE is most affected by residual streaking artifacts and blurry details due to the high undersampling factor. Generally, posterior images of the chest were artifact‐free. For the Cartesian 2D GRE scan, however, anterior images revealed noticeable ghosting, as can be expected from the lack of cardiac‐motion related averaging (see Figure S1). For all volunteers, a highly similar average MTR contrast is observed between all proposed MT sequences, as summarized in Table 1 for selected regions of interest (ROI) in the lung, muscle, and blood (for a definition of ROI, see Figure 1).

**TABLE 1:** Comparison of MTR values for different MT sequences and tissues.

	Lung MTR value (pu) (mean ± SD)	Muscle MTR value (pu) (mean ± SD)	Blood MTR value (pu) (mean ± SD)
3D bSTAR (4)^a^	30.7 ± 1.9	49.6 ± 0.7	20.4 ± 0.9
3D UTE (4)	28.8 ± 1.6	41.6 ± 0.5	17.8 ± 0.9
2D GRE (4)	29.4 ± 2.0	47.7 ± 0.8	18.3 ± 2.5

*Note*: The reported mean and standard deviation (SD) of the MTR were derived from the average MTR value as measured in each volunteer for a region of interest in the tissue (for the definition of the selected regions of interest, see Figure 1).

^a^ The number in round brackets represents the number of volunteers scanned.

Overall, the results presented in Figure 1, Figure S1, and Table 1 demonstrate that MT imaging of the lung appears especially promising with MT‐sensitized bSTAR, offering high‐resolution, artifact‐free, volumetric MTR imaging in free‐breathing and within clinically relevant scan times. Since MT contrast is different for the lung and the blood (see Table 1), however, large blood vessels become visually noticeable as hypointense regions in the high‐resolution MTR contrast images, especially in combination with MT‐sensitized bSTAR (see Figure 1A).

**FIGURE 2:**
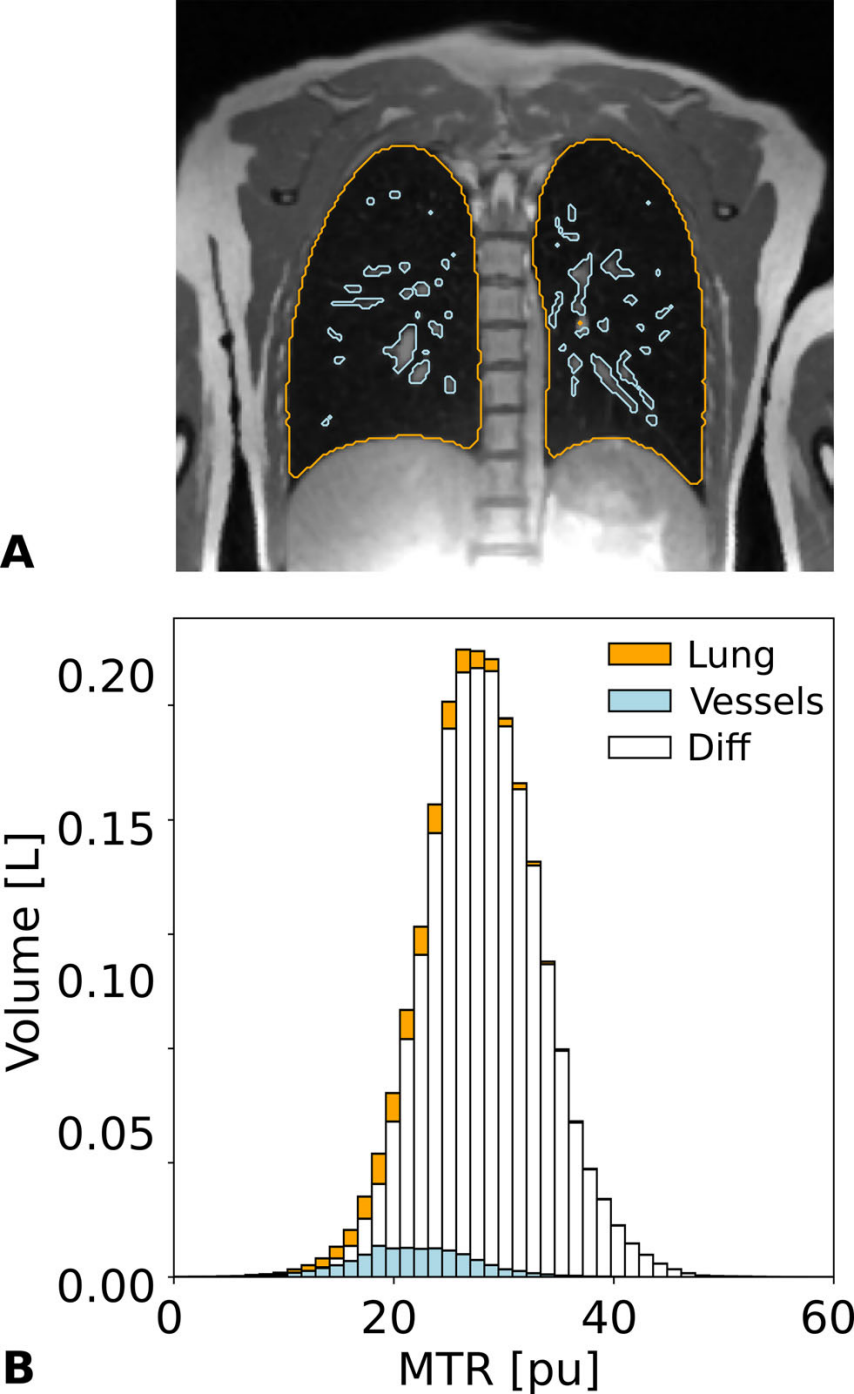
(A) Non‐MT‐weighted coronal bSTAR image of a volunteer. The segmented areas from the lung volume and its vasculature are indicated by the orange (lung) and blue (large vessels) contour lines. (B) MTR histograms for the whole lung (shown in orange) and for the vasculature (shown in blue). To indicate the small volume of vessels, the y axis is measured in volume. The MTR histogram for the parenchyma (shown in white) is given by the difference between the lung and vessel histogram.

The contribution of blood vessels to the MTR of the healthy lung is demonstrated in Figure 2 for one volunteer using a histogram analysis. The segmentation of the lung volume in its visually conspicuous vasculature and remaining lung parenchyma is depicted in Figure 2A for one coronal slice. Corresponding volume MTR histograms are shown in Figure 2B. For the lung, an average MTR value of (28.0 ± 5.5) pu is found, whereas for the vasculature (21.7 ± 5.5) pu is observed, and (28.3 ± 5.2) pu for the lung parenchyma. Although the pulmonary vasculature is pervasive, its total volume fraction within the lung is small (note that to highlight the minimal volumetric impact of vessels, the *y*‐axis of Figure 2B is measured in liters). Consequently, the overall contribution of the vasculature to the MTR histogram is rather negligible and in the following disregarded.

**FIGURE 3:**
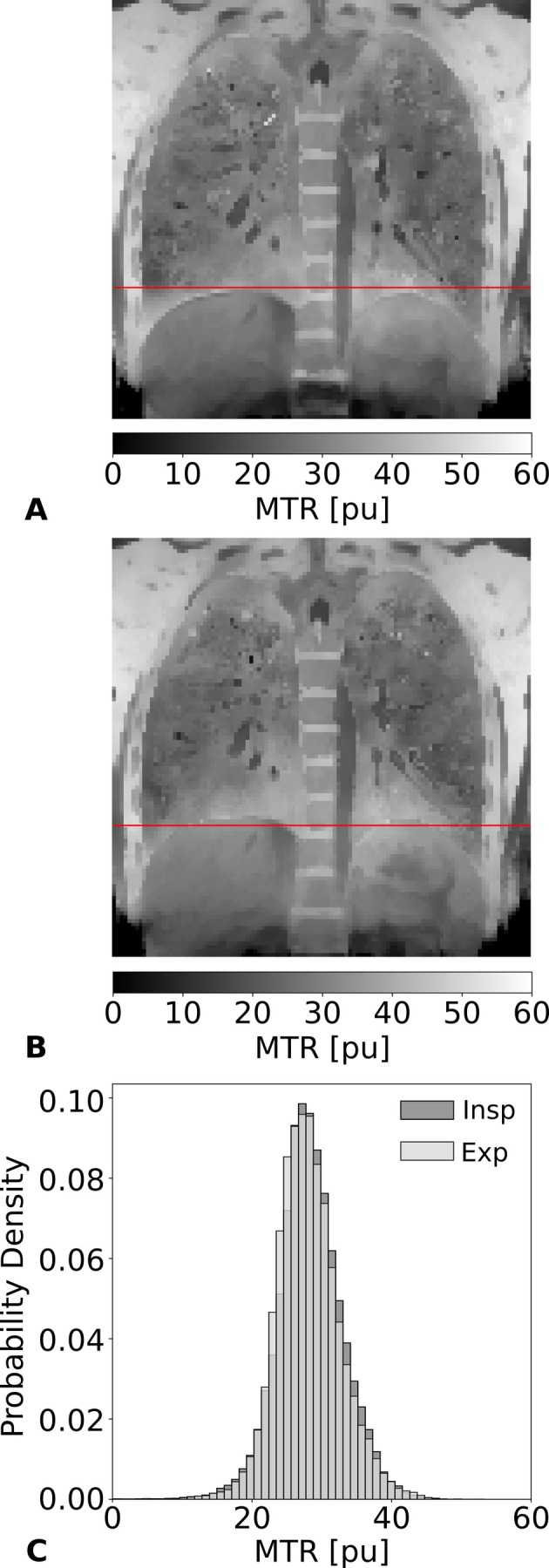
Effect of respiratory volumes on MTR values. (A) Coronal MTR map from the gated inspiration state. (B) Coronal MTR map from the gated expiration state. The red lines mark the diaphragm position in each state. (C) Corresponding whole lung MTR histograms of the inspiration state (dark gray) and the expiration state (light gray).

Generally, self‐gated chest imaging was performed in tidal breathing and images were retrospectively binned to the end‐expiratory tidal phase. Consequently, any change in the tidal breathing depth may lead to small variations of the reconstructed end‐expiratory tidal lung volume which might affect lung MTR measurements. This possible effect is investigated in Figure 3 for MT‐sensitized bSTAR. Example coronal MTR images are shown in Figure 3A in the inspiratory tidal phase and in Figure 3B in the expiratory tidal phase. Corresponding MTR histograms are shown in Figure 3C with a mean of 28.3 pu for the inspiratory and 28.0 pu for the expiratory case. Overall, a relative change of 8% in the lung volume, derived from the respective lung masks created for both scans, resulted in only a subtle change of 0.3 pu in its mean MTR.

Finally, the long‐term intra‐individual reproducibility and inter‐individual variability for MTR imaging with MT‐sensitized bSTAR is assessed from a scan‐rescan experiment performed in volunteers. For all volunteers, a low intra‐individual variability for the mean MTR value was observed (with a coefficient of variation ranging from 6% to 9%), indicating good reproducibility. Moreover, the small group of young volunteers with similar age revealed highly similar inter‐individual MTR values for the lung of (29.6 ± 2.4) pu. For a detailed summary of the results, see Table 2. Moreover, whole lung MTR histograms of the normal lung appear to follow a Gaussian distribution and thus exhibit an overall symmetric shape for individual subjects, as well as for the collective of healthy volunteers (see Figure S2).

**TABLE 2:** Reproducibility of average whole lung MTR values in healthy volunteers.

	MTR value (pu) (mean ± SD)
Volunteer 1 (5)^a^	28.8 ± 2.5
Volunteer 2 (5)	30.6 ± 1.9
Volunteer 3 (5)	29.2 ± 2.5
Volunteer 4 (5)	29.6 ± 2.1

*Note*: The reported mean and standard deviation (SD) of the MTR were derived from the average whole lung MTR values observed in the individual scan‐rescan experiment.

^a^ The number in round brackets represents the number of scan‐rescan tests used.

In the following, the feasibility and prospects of whole lung MTR imaging with MT‐sensitized bSTAR are further analyzed and illustrated in the clinical setting using example patient cases with known lung disease.

**FIGURE 4:**
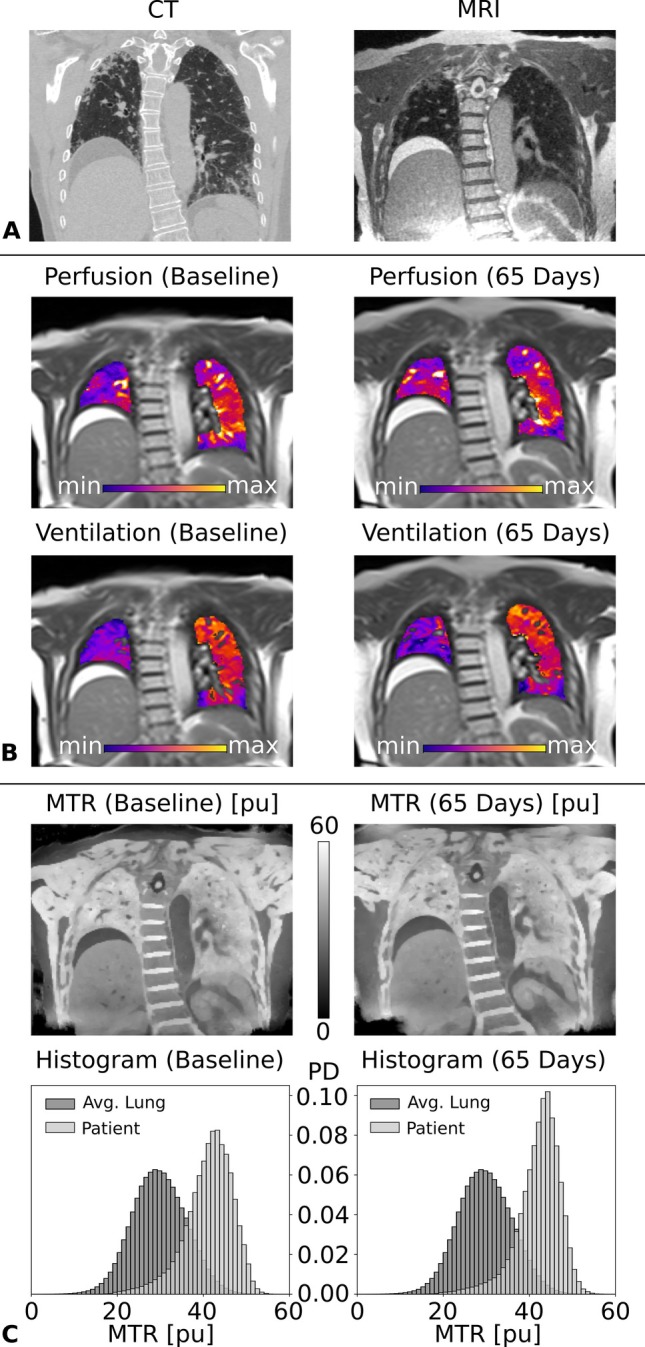
Pulmonary fibrosis patient with acute inflammation: (A) morphological images: CT and MRI, (B) functional maps: perfusion‐weighted MP MRI map and ventilation‐weighted MP MRI map of the first visit on the left and of the second visit after 65 days on the right, and (C) MTR maps and whole lung MTR probability density (PD) histograms in light gray of the first visit on the left and of the second visit after 65 days on the right. For comparison, a histogram representing collective data of all healthy volunteers, representing an average healthy lung, is shown in dark gray.

The imaging results for a patient (75 years, female) diagnosed with fibrosing hypersensitivity pneumonitis are collected in Figure 4. The patient presented with severe restrictive ventilation impairment and severely reduced hemoglobin‐corrected diffusing capacity for carbon monoxide. The chest CT (Figure 4A left) and the bSTAR (Figure 4A right) revealed localized fibrotic regions in the lung. The patient underwent bronchoalveolar lavage (BAL) which showed a lymphocytosis of 38% (normal range: 5%–15%), consistent with acute inflammation. Two MT scans were acquired in two visits 65 days apart, the first prior to BAL. Corresponding lung perfusion (Figure 4B, top) and ventilation (Figure 4B, bottom) maps from MP MRI clearly indicate a strong functional impairment in the right lung, as well as hypoperfused and hypoventilated regions affected by fibrosis in the lower lobe of the left lung. No significant change in ventilation and perfusion can be detected between the two visits. The MTR maps (Figure 4C, top) show a similar visual appearance for the right and left lung but are overall shifted towards higher MTR values (mean MTR of 41.2 pu for the first, and 42.2 pu for the second visit) in comparison to a collective dataset of healthy volunteers. Like the functional maps, the two MT maps and the corresponding histograms (Figure 4C, bottom) are close to identical.

**FIGURE 5:**
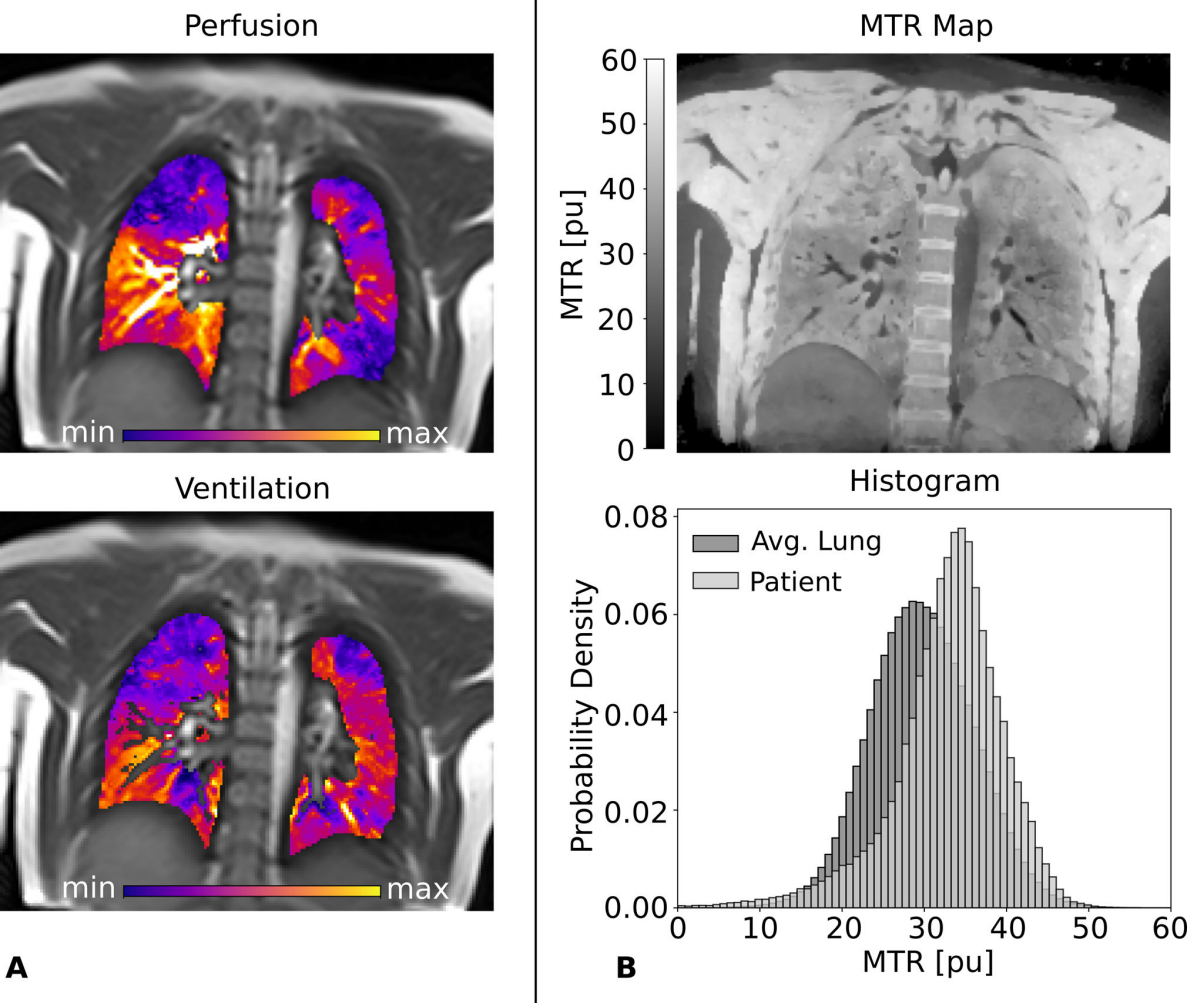
Cystic fibrosis patient: (A) Perfusion‐weighted MP MRI map and ventilation‐weighted MP MRI map and (B) MTR map and whole lung MTR histogram for the patient in light gray and collective data of healthy volunteers, representing an average healthy lung, in dark gray.

Figure 5 shows the imaging results for a cystic fibrosis patient (22 years, female) who came in for a regular visit. In the bSTAR image, bronchiectasis with mucus plugging was found in the right upper lobe. MRI‐based lung perfusion (Figure 5A, top) and ventilation (Figure 5A, bottom) maps indicate corresponding impairments. The impairments in the upper right lung are also clearly visible in the MTR map (Figure 5B, top), with MTR values of around 39.8 pu compared to values of 32.2 pu in the lower right lung in the depicted slice. MTR values in the overall lung are higher than in healthy volunteers, with a mean MTR of 32.8 pu (Figure 5B, bottom).

**FIGURE 6:**
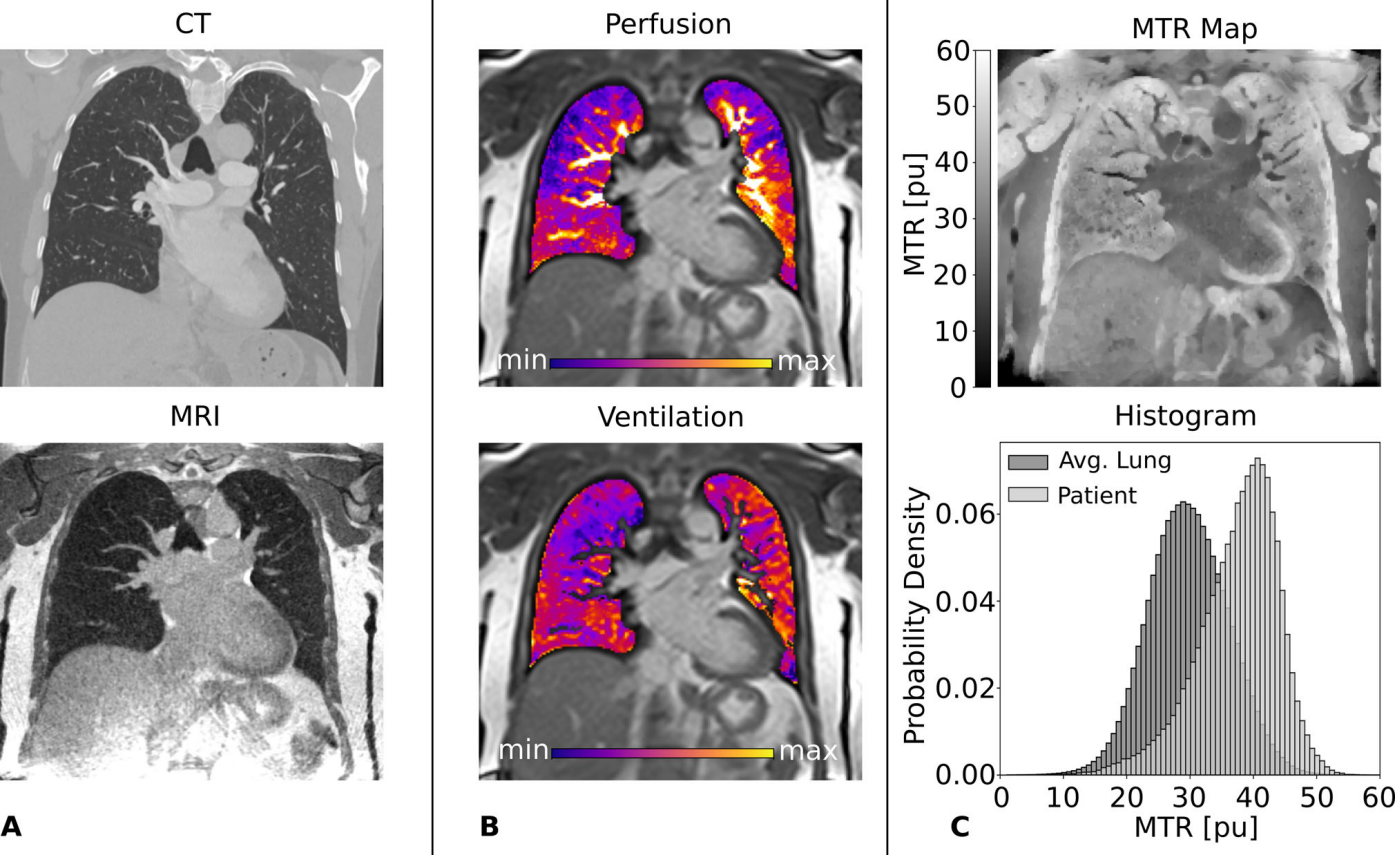
Patient with T2‐low asthma: (A) Morphological images: CT and MRI, (B) functional maps: perfusion‐weighted MP MRI map and ventilation‐weighted MP MRI map, and (C) MTR maps and whole lung MTR histogram for the patient in light gray and collective data of healthy volunteers, representing an average healthy lung, in dark gray.

In a third case, a 52‐year‐old female patient with T2‐low asthma, structural imaging via CT (Figure 6A, top) and bSTAR (Figure 6A, bottom) was unremarkable. Nevertheless, the lung perfusion (Figure 6B, top) and ventilation (Figure 6B, bottom) maps from MP MRI indicate a functional impairment in the upper lung. Corresponding results are revealed by the MTR map (Figure 6C, top), where MTR values are elevated in the upper lung. The whole lung MTR histogram (Figure 6C, bottom) reveals a generally higher MTR distribution than in healthy volunteers with a mean MTR of 37.6 pu.

**FIGURE 7:**
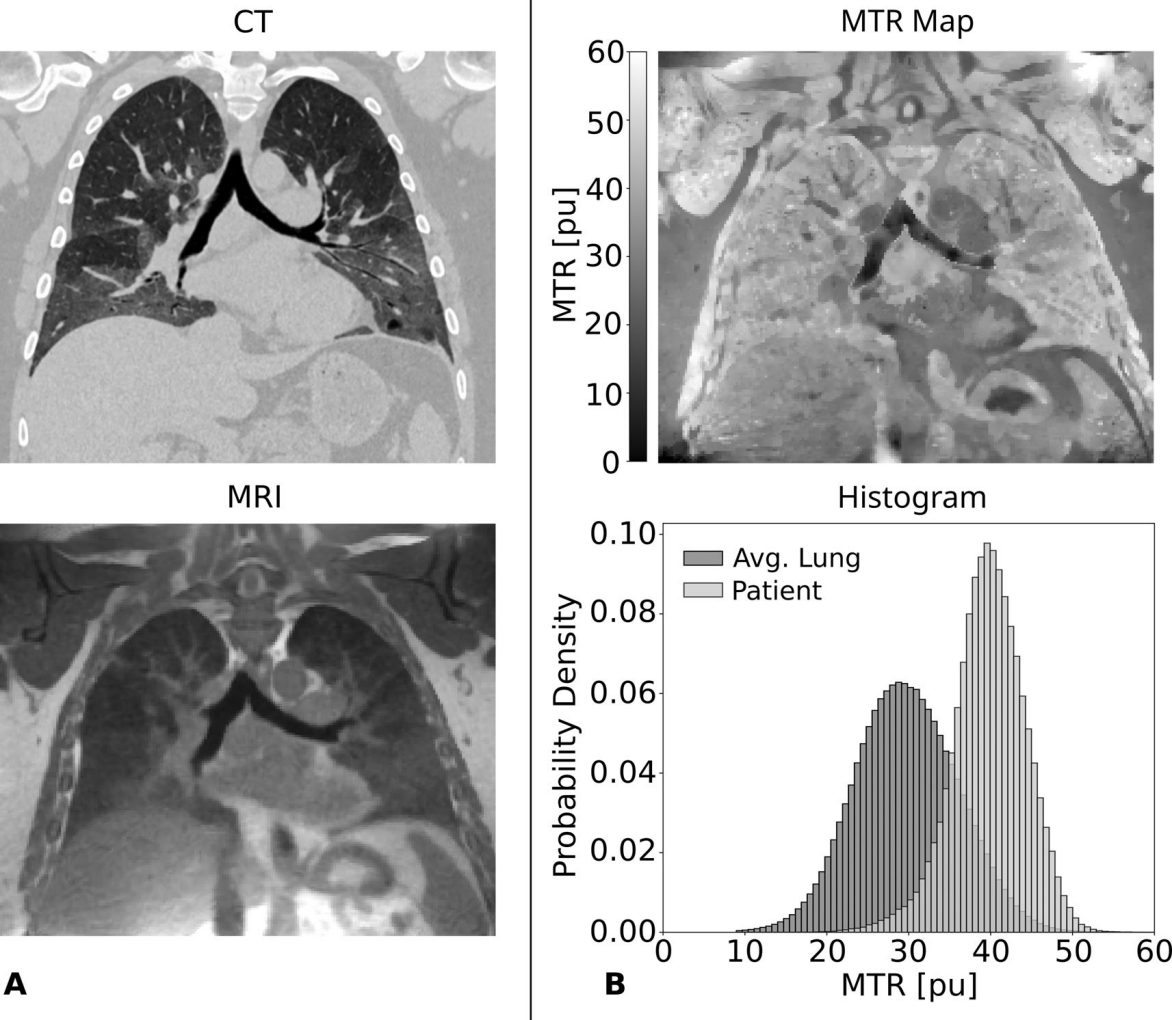
Patient with chronic thromboembolic pulmonary hypertension (CTEPH): (A) morphological images: CT and MRI, and (B) MTR maps and whole lung MTR histogram for the patient in light gray and collective data of healthy volunteers, representing an average healthy lung, in dark gray.

Finally, the imaging results for a patient (40 years, male) with chronic thromboembolic pulmonary hypertension (CTEPH) are presented in Figure 7. Corresponding to ground‐glass opacity in CT (Figure 7A, top), hyperintense areas can be seen in the bSTAR image (Figure 7A, bottom), but without noticeable contrast in the MTR image (Figure 7B, top). Overall, higher MTR values with a mean MTR of 39.8 pu are observed as compared to healthy volunteers (Figure 7B, bottom).

To summarize, Table 3 shows the mean lung MTR for the studied pathologies as compared to an average healthy lung estimated from the four healthy volunteers. Again, MTR values are generally much higher in patients with pulmonary disease compared to healthy volunteers.

**TABLE 3:** Whole lung average MTR in diseased and normal lung.

	MTR value (pu)
Normal lung (4)^a^	29.6 ± 2.4
Pulmonary fibrosis (4)	40.6 ± 0.7
Cystic fibrosis (1)	32.8
T2‐low asthma (1)	37.6
CTEPH (1)	39.8

*Note*: The reported mean and standard deviation of the MTR for normal lung were calculated from the reported average whole lung MTR values given in Table 2. The reported mean and standard deviation of the MTR for the pulmonary fibrosis patients were derived from the average whole lung MTR values of each patient. The reported MTR values for the other patients refer to the whole lung average MTR value.

^a^ The number in round brackets represents the number of volunteers or patients with specific diseases scanned.

## 4 Discussion

The possibility of in vivo MT MRI of the human lung for detecting a wide range of parenchymal tissue abnormalities at low fields was studied by a single pioneering original paper from 1995 that was not subsequently pursued in the literature [3]. At that time, the clinical prospects of the proposed method were likely hampered by the limited hardware performance and availability of 0.1 T MRI scanners. Furthermore, the clinical acceptability of a single‐slice method with an overall scan time of approximately 13 min may have been considered inadequate.

From the above‐mentioned historical lack of suitable MT sequences for the lung, first the feasibility of MTR imaging on a commercial 0.55 T scanner with state‐of‐the‐art hardware and imaging sequences was evaluated in a group of volunteers. In this work, two custom self‐gated 3D MT imaging sequences were developed and implemented for free‐breathing MTR imaging of the lung using either MT‐sensitized bSTAR or MT‐prepared UTE, whereas vendor‐provided MT imaging was only possible in 2D and in breath‐holding using MT‐prepared GRE. Overall, a highly similar MT contrast was observed for all investigated MT sequences, irrespective of the different methods used for MT contrast generation.

The achievable resolution, however, was notably different between the methods. It is highest for MT‐sensitized bSTAR, but about four times smaller for MT‐prepared UTE and about 18 times smaller for MT‐prepared GRE. Moreover, MT‐prepared UTE imaging required clinically unfeasible scan times, and 2D MT‐prepared GRE, whilst being theoretically fast, required in practice a series of repeated breath‐holding maneuvers to resolve a possible mismatch in breath‐holding positions that would otherwise mask MT‐related signal differences. Furthermore, 2D MT‐prepared GRE may suffer from cardiac motion‐related image artifacts. In contrast, self‐gated sequences generally rely on steady breathing cycles and from the rather long scan time requirements can be prone to intra‐scan motion, especially in patients or children. No dropouts, however, were observed for the limited number of patient scans performed so far.

Overall, MT‐sensitized bSTAR clearly outperforms both MT‐prepared UTE and GRE methods at 0.55 T, but it is important to note that bSSFP‐based MTR mapping can be affected by B0 field inhomogeneities [30]. RF pulse prolongation is required to reduce MT‐weighting, which inevitably also prolongs the TR. Generally, proper shimming of the lung might help to mitigate bSSFP's off‐resonance sensitivity but the required upper range in the TR (about 3 ms) is likely far too long for artifact‐free bSSFP MRI of the chest at fields higher than 0.55 T (since even in combination with a high‐end gradient system, the upper boundary of the TR will not be substantially shortened). Thus, low‐field systems appear to be mandatory for MTR mapping of the lung with the proposed methodology. Thus, currently, transferability of the proposed method to other sites is limited by the availability of low field MRI scanners in the clinic.

In summary, MTR mapping of the lung with MT‐sensitized bSTAR indicated not only a high long‐term intra‐individual reproducibility for the average whole lung MTR value for a group of volunteers with similar age (as well as in a representative patient with chronic lung disease) but also an overall low inter‐individual variability. Our results also demonstrate that pulmonary diseases, such as pulmonary fibrosis, cystic fibrosis, T2‐low asthma, and CTEPH, can affect MT contrast, as initially observed by Kuzo et al. [3] and that they lie within the expected range [3, 4]. Furthermore, since MT is sensitive to changes in the bound proton pool, such as changes in protein content, MTR may enable the detection of early fibrotic processes that are not directly visible using structural MRI techniques, as already pointed out by Kuzo et al. [3].

Generally, the patients not only showed overall higher MTR values but also a left skewed MTR distribution compared to the healthy control group. MTR histogram analysis is frequently performed in the brain to assess pathologies or tissue abnormalities; especially for multiple sclerosis [31]. For development and validation of such lung MTR histogram metrics, however, clinical studies will be required.

A current limitation of our study is the lack of an age‐matched control group and the limited patient cohort. As compared to the group of healthy young volunteers, substantially elevated mean MTR values were found in the studied patients. Generally, pulmonary structure and function have been shown to change with increasing age using hyperpolarized ^129^Xe MRI [32, 33, 34]. Thus, age could be a possible important covariate for the development of MTR histogram metrics as mentioned above. Besides clinical studies, future work will also need to investigate a possible age‐dependency of normative MTR values.

## 5 Conclusions

Artifact‐free MTR imaging of the whole lung becomes feasible in clinically relevant scan times and in free‐breathing at 0.55 T using MT‐sensitized bSTAR and demonstrated high reproducibility in volunteers and high sensitivity to parenchymal tissue alterations in patients. This may open up new perspectives for a non‐invasive investigation and differentiation of lung diseases beyond current structural and functional assessments. Generally, successful MT‐sensitized bSTAR imaging, however, requires excellent field homogeneities which—at present—appear to be granted only for a currently emerging new generation of low‐field MRI systems, as used in this work.

## Funding

This work was supported by Swiss National Science Foundation, 320030_219186.

## Conflicts of Interest

Jonathan Röcken received payment for a presentation from Astra‐Zeneca AG and received support for attending a congress from Astra‐Zeneca AG and OM‐Pharma. The other authors declare no conflicts of interest.

## Supporting information


**Table S1:** Sequence parameters for the three investigated sequences for MTR imaging. *Unconventional MT prep for bSSFP kernels refers to a modulation of the duration of the RF excitation pulse. MT‐weighting used a hard RF pulse of 100 μs duration, non‐MT‐weighting used a hard pulse of 1500 μs duration. Conventional MT prep for RF spoiled GRE refers to pulsed off‐resonance irradiation with a nonselective Gaussian RF pulse (7680 μs duration at 1500 Hz) having a flip angle of 500°. **Including a resting period after each breath‐hold of equal duration (6 × 0:24 min + 5 × 0:24 min).
**Figure S1:** Sequence comparison for an example coronal slice with non‐MT‐weighted images (left column), MT‐weighted images (middle column), and resulting MTR maps (right column) for 3D bSTAR (A), 3D UTE (B), and 2D GRE (C). The artifacts in the 2D GRE MTR map result from cardiac motion (see red arrows).
**Figure S2:** (A) Average MTR histogram representing the collective data of all five scans from all four healthy volunteers with a Gaussian fit (black line). (B) Single scan MTR histogram of an example healthy volunteer with a Gaussian fit (black line). Both plots indicate the near Gaussian shape of the MTR distribution.

## Data Availability

The data that support the findings of this study are available from the corresponding author upon reasonable request.
