# Supplementary material for: Free‐Breathing Magnetization Transfer Imaging of the Lung at 0.55 T Using bSTAR
Source: Magn Reson Med. 2026 Mar 12;96(1):203–13. doi: 10.1002/mrm.70341 (PMC13156434; doi:10.1002/mrm.70341)
Supplement: Supplementary file 1 — Table S1: Sequence parameters for the three investigated sequences for MTR imaging. *Unconventional MT prep for bSSFP kernels refers to a modulation of the duration of the RF excitation pulse. MT‐weighting used a hard RF pulse of 100 μs duration, non‐MT‐weighting used a hard pulse of 1500 μs duration. Conventional MT prep for RF spoiled GRE refers to pulsed off‐resonance irradiation with a nonselective Gaussian RF pulse (7680 μs duration at 1500 Hz) having a flip angle of 500°. **Including a resting period after each breath‐hold of equal duration (6 × 0:24 min + 5 × 0:24 min). Figure S1: Sequence comparison for an example coronal slice with non‐MT‐weighted images (left column), MT‐weighted images (middle column), and resulting MTR maps (right column) for 3D bSTAR (A), 3D UTE (B), and 2D GRE (C). The artifacts in the 2D GRE MTR map result from cardiac motion (see red arrows). Figure S2: (A) Average MTR histogram representing the collective data of all five scans from all four healthy volunteers with a Gaussian fit (black line). (B) Single scan MTR histogram of an example healthy volunteer with a Gaussian fit (black line). Both plots indicate the near Gaussian shape of the MTR distribution. [file MRM-96-203-s001.docx]

**SUPPORTING INFORMATION**

| Sequence | bSTAR (custom) | UTE (custom) | GRE (product) |
| --- | --- | --- | --- |
| Dimension | 3D | 3D | 2D multi-slice |
| Acquisition | Radial | Radial | Cartesian |
| FOV [mm] | 340x340x340 | 370x370x370 | 450x450 |
| Resolution [mm] | 1.9x1.9x1.9 | 3.0x3.0x3.0 | 3.5x3.5 |
| Slice thickness [mm] | -- | -- | 10 |
| Echo Type | dual half-echo | single half-echo | single echo |
| Bandwidth [Hz/Px] | 1235 / 1235 | 401 | 260 |
| Gating | self-navigated | self-navigated | breath-hold |
| Spokes | 100000 | 28000 | -- |
| Interleaves | 500 | 1400 | -- |
| Flip angle [deg] | 30 | 8 | 30 |
| MT Prep* | unconventional | conventional | conventional |
| TE (MT) [ms] | 0.08 / 1.70 | 0.08 | 3.59 |
| TE (noMT) [ms] | 0.78 / 2.40 | 0.08 | 3.59 |
| TR (MT) [ms] | 1.86 | 18.5 | 315 |
| TR (noMT) [ms] | 3.26 | 18.5 | 315 |
| Scan time (MT) [min] | 3:06 | 8:38 | 3 x 0:24 |
| Scan time (noMT) [min] | 5:26 | 8:38 | 3 x 0:24 |
| Total Time [min] | 8:32 | 17:16 | approx. 5:00** |

**Table S1:** Sequence parameters for the three investigated sequences for MTR imaging. *Unconventional MT prep for bSSFP kernels refers to a modulation of the duration of the RF excitation pulse. MT-weighting used a hard RF pulse of 100 µs duration, non-MT-weighting used a hard pulse of 1500 µs duration. Conventional MT prep for RF spoiled GRE refers to pulsed off-resonance irradiation with a non-selective Gaussian RF pulse (7680 µs duration at 1500 Hz) having a flip angle of 500°. **Including a resting period after each breath-hold of equal duration (6 x 0:24 min + 5 x 0:24 min).


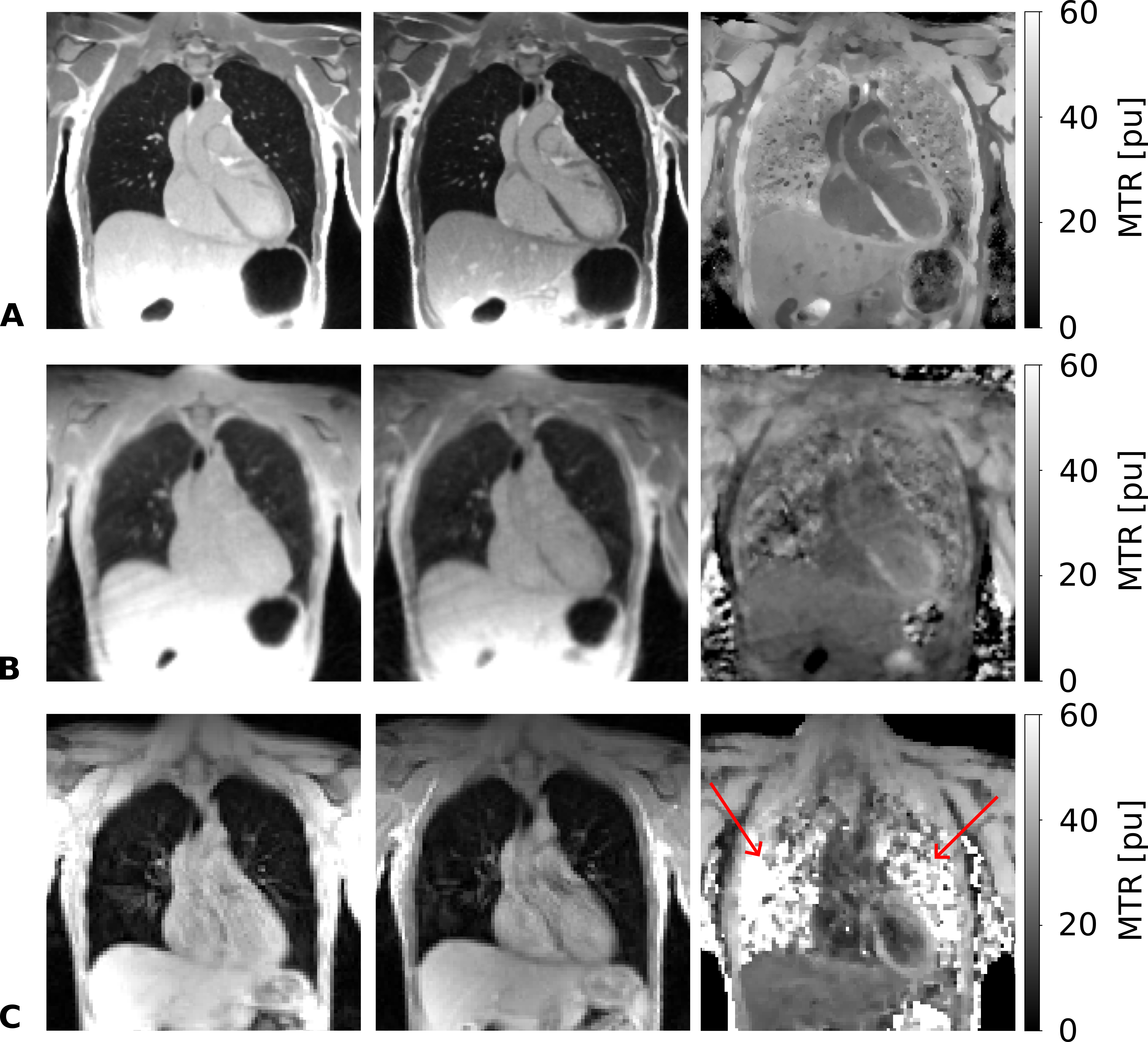
**Figure S1:** Sequence comparison for an example coronal slice with non-MT-weighted images (left column), MT-weighted images (middle column), and resulting MTR maps (right column) for 3D bSTAR (A), 3D UTE (B), and 2D GRE (C). The artifacts in the 2D GRE MTR map result from cardiac motion (see red arrows).


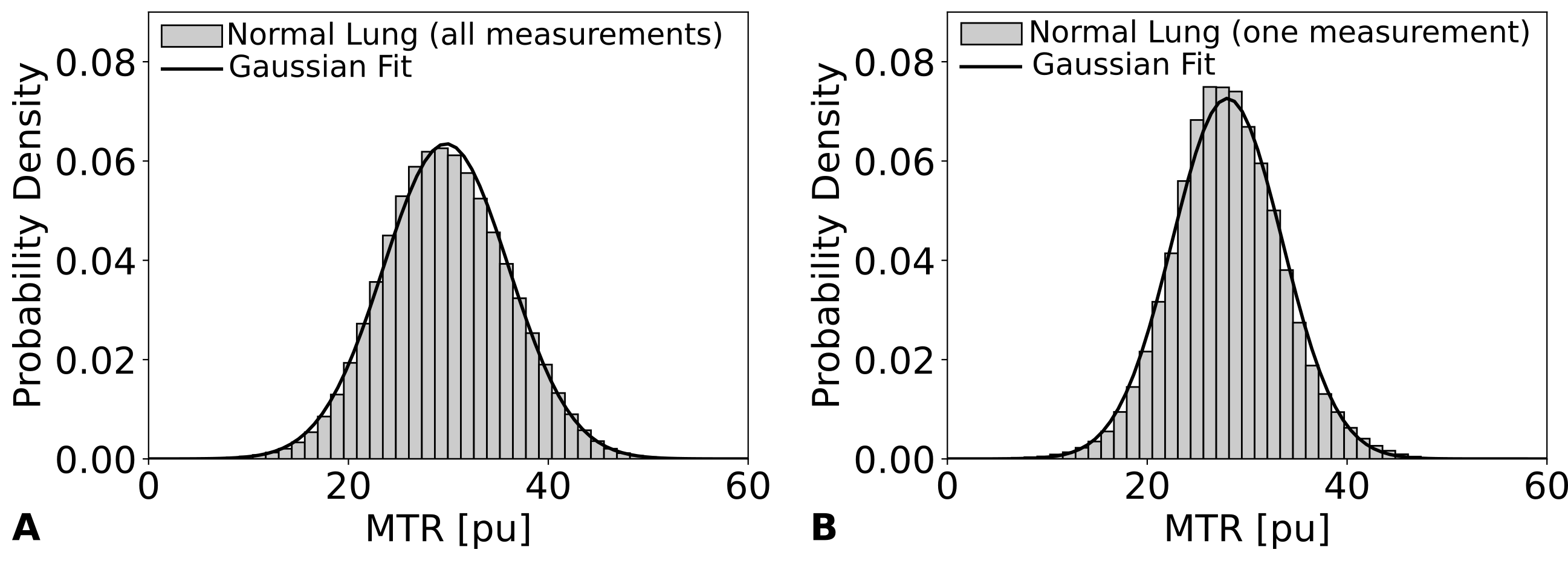
**Figure S2:** (A) Average MTR histogram representing the collective data of all five scans from all four healthy volunteers with a Gaussian fit (black line). (B) Single scan MTR histogram of an example healthy volunteer with a Gaussian fit (black line). Both plots indicate the near Gaussian shape of the MTR distribution.
